# Female Sex Hormones and Cardiac Pressure Overload Independently Contribute to the Cardiogenic Dementia Profile in Yucatan Miniature Swine

**DOI:** 10.3389/fcvm.2019.00129

**Published:** 2019-09-10

**Authors:** Grant C. Hayward, Paul J. LeBlanc, Craig A. Emter, Jennifer N. K. Nyarko, Darrell D. Mousseau, Rebecca E. K. MacPherson, T. Dylan Olver

**Affiliations:** ^1^Department of Health Sciences, Brock University, St. Catharines, ON, Canada; ^2^Centre for Neuroscience, Brock University, St. Catharines, ON, Canada; ^3^Centre for Bone and Muscle Health, Brock University, St. Catharines, ON, Canada; ^4^Department of Biomedical Sciences, University of Missouri, Columbia, MO, United States; ^5^Department of Psychiatry, College of Medicine, University of Saskatchewan, Saskatoon, SK, Canada; ^6^Department of Biomedical Sciences, Western College of Veterinary Medicine, University of Saskatchewan, Saskatoon, SK, Canada

**Keywords:** swine, heart failure, female sex hormones, dementia, brain

## Abstract

Post-menopausal women with heart failure (HF) frequently exhibit cardiogenic dementia. Using a pre-clinical swine model of post-menopausal HF, we recently demonstrated that experimental menopause (ovariectomy; OVX) and HF (6-month cardiac pressure overload/aortic banding; AB) independently altered cerebral vasomotor control and together impaired cognitive function. The purpose of this study was to examine the prefrontal cortex and hippocampus tissues from these animals to assess whether OVX and HF are associated with neurologic alterations that may contribute to cardiogenic dementia. We hypothesized that OVX and HF would independently alter neuronal cell signaling in swine with post-menopausal cardiogenic dementia. Immunoblot analyses revealed OVX was associated with reduced estrogen receptor-α in both brain regions and HF tended to exacerbate OVX-induced deficits in the hippocampus. Further, OVX was associated with a reduction in the ratio of phosphorylated:total Akt and ERK in the hippocampus as well as decreased total Akt and synaptophysin in the prefrontal cortex. In contrast, HF was associated with a trend toward reduced phosphorylated:total ERK in the prefrontal cortex. In addition, HF was associated with decreased β-amyloid (1–38) in the prefrontal cortex and increased β-amyloid (1–38) in the hippocampus. Regional brain lipid analysis revealed OVX tended to increase total, saturated, and monounsaturated fatty acid content in the prefrontal cortex, with the greatest magnitude of change occurring in the AB-OVX group. The data from this study suggest that OVX and HF are independently associated with regional-specific neurologic changes in the brain that contribute to the cardiogenic dementia profile in this model. This pre-clinical swine model may be a useful tool for better understanding post-menopausal cardiogenic dementia pathology and developing novel therapies.

## Introduction

The term “cardiogenic dementia” describes the association between the failing heart and the failing mind (1). Cardiogenic dementia is associated with reduced quality of life, increased hospital readmission, and risk of mortality (2–5). In the setting of heart failure with preserved ejection fraction (HFpEF; HF subtype where EF% is maintained ≥50%), it is estimated up to 50% of patients experience cardiogenic dementia (6, 7). Of note, HFpEF disproportionally affects older women (~2:1 vs. men) implicating the loss of female sex hormones in the onset of the disease (8). The underlying causes of cognitive impairment in HFpEF are difficult to elucidate as dementia phenotypes are non-uniform in this population (9). For example, cardiogenic dementia may reflect aspects of Alzheimer's disease (AD), vascular dementia, frontotemporal dementia, or some combination of these (9). Potentially, the development of HF as well as any loss of sex hormones, e.g., with menopause, could contribute to the cardiogenic dementia phenotype in female patients.

Recently, using a pre-clinical model of experimental HF (cardiac pressure overload) that exhibits cardiac features consistent with HFpEF in male swine, we observed that induction of HF alone coincided with increased carotid artery vascular resistance, impairments in cranial blood flow control, and deficits in memory performance, independent of resting cardiac systolic impairment (10). In a follow-up study using the same model of HF, we examined the effect of experimental menopause (ovariectomy) on indices of cardiogenic/vascular dementia in female swine and demonstrated that HF alone led to impairments in cerebrovascular function (11). Further, the loss of female sex hormones exacerbated impairments in brain blood flow control as well as deficits in memory performance. Collectively, the data implicate independent and interactive roles of HF and the loss of female sex hormones in the development of vascular indices of cardiogenic dementia and support the view that HFpEF is a total-body syndrome affecting the peripheral vasculature and the brain (6, 7, 9, 12).

Two brain regions involved in the encoding and retrieval of memories are the prefrontal cortex and the hippocampus (13). Of note, HF appears to be a contributing factor to AD, and in particular to AD pathology in prefrontal cortex and hippocampal regions (14). Two major histopathological hallmarks of AD are senile plaques, resulting from accumulations of β-amyloid (Aβ) peptides, and neurofibrillary tangles, which are a result of hyperphosphorylated tau protein (15–17). The pathophysiology of adverse neural outcomes in the setting of HF is unclear, but may be a primary adaptation or occur secondary to HF-related cerebral hypoperfusion, cerebro-microvascular dysfunction or some combination of these factors. The loss of female sex hormones may also be an independent contributor to cardiogenic dementia in older women, independent of HF (18–20). Emerging evidence indicates a role for estrogen receptor-α (ERα) signaling in central lipid homeostasis (21–23) and a recent report documented increased brain lipid content in brain biopsies from deceased patients with late-stage AD (24). In addition to disturbances in the brain lipid profile, both mitogen activated protein kinase/extracellular signal-regulated kinase (MAPK/ERK) and protein kinase B (PKB aka Akt) signaling are involved in the encoding and retrieval of memories. Dysregulation of these two signaling pathways is associated with deficits in cognition, synaptic plasticity, and neuronal survival (23, 25–28), raising the possibility that HF and the loss of female sex hormones may alter regulation of these key signaling pathways, independently or in a synergistic manner.

Herein, we performed a retrospective analysis on prefrontal cortex and hippocampal tissues from the aforementioned female swine study (11). The purpose of this analysis was to examine the independent and interactive effects of HF (cardiac pressure overload) and the loss of female sex hormones (ovariectomy) on estrogen receptor content, ERK and Akt signaling as well as on selected molecular markers of AD. We hypothesized that both HF and the loss of female sex hormones would alter indices of ERK and Akt signaling as well as Aβ peptide levels in swine with cardiogenic dementia. In addition, we examined brain lipid profiles to determine whether the loss of female sex hormones and HF would increase lipid accumulation in the prefrontal cortex and hippocampus.

## Methods

### Animals and Design

**Figure 1:**
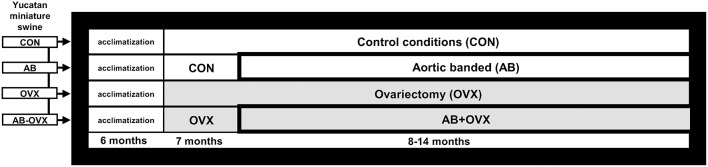
Study design schematic. Timeline of ovariectomy and aortic banding surgeries. Euthanasia was completed for all groups at 14 months. CON, control; AB, aortic banding; OVX, ovariectomy; AB + OVX, aortic banding + ovariectomy.

This was a retrospective analysis on tissues harvested from swine used in a study published previously (11) where all experimental and animal protocols were approved by the University of Missouri (Columbia, MO) Animal Care and Use Committee (Protocol #8907). The experimental design and animal characteristics, including cognitive function and cardiac phenotype, have been described previously (11). Briefly, sexually mature Yucatan miniature swine (7 months old; *N* = 28) were divided into four groups: intact control (CON), intact aortic banding (AB), non-AB ovariectomy (OVX), or AB-OVX (*n* = 7 per group). Experimental OVX is a common *in vivo* model used to recapitulate the loss of female sex hormones with aging (29) and AB was used as a cardiac pressure overload model of HF. The OVX and AB surgeries were performed at 7 and 8 months, respectively (Figure 1).

#### Ovariectomies [Described in Our Recent Report (11)]

At 7 months of age, the OVX and AB-OVX groups were sedated with telazol/xylazine (5 and 2.25 mg·kg^−1^, respectively) and maintained under anesthesia with 3.0% isoflurane while the ovaries were removed (30). One month following ovariectomy serum progesterone (chemiluminescent enzyme immunoassay; IMMULITE 1000) was not detectable in OVX and AB-OVX groups and at the end of the study, as expected with the loss of female sex hormones, uterine mass was ~10 times lower in the OVX and AB-OVX groups compared to intact (CON and AB).

#### Aortic Banding [Described in Our Recent Report (11)]

At 8 months of age, AB, and AB-OVX groups underwent AB surgeries as previously reported by our laboratory (10, 11). The aortic band was placed around the ascending aorta and a systolic trans-stenotic gradient of ~70 mmHg was achieved (AB = 73 ± 3 and AB-OVX = 72 ± 3 mmHg) under equivalent hemodynamic conditions for all pigs (heart rate, AB = 99 ± 5 and AB-OVX = 109 ± 4 bpm; mean arterial pressure = AB = 90 ± 1 and AB-OVX = 89 ± 1 mmHg).

#### Altrenogest Dosing [Described in Our Recent Report (11)]

To account for menstruation and ensure the intact swine were not in estrus at the time of experimentation, menstrual cycles were synchronized. Briefly, the intact swine were dosed orally with the steroidal progestin, altrenogest (4.5 mL, 0.22% solution; MATRIX^®^, Merck, New Jersey) for 14 days, followed by 12–15 days of non-treatment before testing (confirmed as circulating progesterone >0.5 ng/mL at end point procedures) (31, 32).

### Tissue Collection and Western Blot Analyses

At 14 months of age, swine were anesthetized using a mixture of Telazol/xylazine (5 and 2.25 mg/kg, respectively) euthanized by exsanguination. Thereafter, tissue samples from the left and right hippocampus and prefrontal cortex were quickly removed, snap frozen in liquid nitrogen, and stored at −80°C until further analyses (33). To perform Western (immuno)blotting, brain tissue was homogenized (FastPrep, MP Biomedicals, Santa Ana, CA) in 20 volumes of NP40 Cell Lysis Buffer (Life Technologies; cat# FNN0021) containing protease (Sigma-Aldrich, 11836170001) and phosphatase inhibitors (Sigma-Aldrich, 04906845001). Samples were then centrifuged at 4°C for 15 min at 10,000 × g, and the supernatant was collected. Protein concentrations were determined using BCA quantification assay (34). Samples were prepared using 2x Laemmli buffer and total protein concentrations were equalized. Twenty microgram of protein was resolved on 10% SDS PAGE gels (1.5 h at 120 V). Proteins were then wet transferred to nitrocellulose membranes (GE Healthcare Life Science; cat#10600002) for 1 h at 100 V, followed by blocking in 5% milk casein in Tris-buffered saline/0.1% Tween 20 (TBST) for 1 h. Membranes were incubated overnight on a shaker with primary antibody and then rinsed with TBST, and incubated for 1.5 h with appropriate horse-radish peroxidase-conjugated secondary antibodies (1:1,000; Jackson ImmunoResearch Laboratories, West Grove, PA) at room temperature. A representative β-actin immunoblot was measured and analyzed for each membrane to ensure equal loading (<10% variability across gels and no between group differences were observed). Signals were detected using enhanced chemiluminesence Western lightning Plus-ELC (PerkinElmer, 105001EA) and were subsequently quantified by densitometry using a FluorChem HD imaging system (Alpha Innotech, Santa Clara, CA). Protein markers included ERα (1:500; Santa Cruz cat# sc-8005; molecular weight (MW) = 66 kDa), ERK (1:1,000; Cell Signaling cat #4695S; MW = 42,44 kDa), pERK (1:500; Cell Signaling cat #9101S; MW = 42,44 kDa), Akt (1:1,000; Cell Signaling cat #4685S; MW = 60 kDa), and pAkt Ser473 (1:250; Cell Signaling cat #4058S MW = 60 kDa), as well as a marker of downstream PI3K/Akt, e.g., glycogen synthase kinase (GSK3β; 1:1,000; Cell Signaling cat #9315S; MW = 46 kDa), pGSKβ Ser 9 (1:500; Cell Signaling cat #5558S; MW = 46 kDa), pTau Ser396 (1:5,000; Abcam cat# ab109390; MW 55 kDa), insulin degrading enzyme (IDE; 1:500, Santa Cruz cat #sc-393887; MW = 118 kDa), and synaptophysin (1:1,000; Cell Signaling cat #5461; MW = 38 kDa).

### Immunoprecipitation for Alzheimer Disease (AD)-Related Markers

The 6E10 antibody [targets Aβ(1–16): cat# SIG-39320] and the 4G8 antibody [targets Aβ(17–24): cat# SIG-39220] were obtained from Cedarlane Laboratories Ltd. The antibody raised against the C-terminal region of human APP695 (amino acids 676–695: cat# A8717) was obtained from Sigma-Aldrich. Cortical and hippocampal samples (20–30 mg wet weight) were homogenized in 20 volumes of ice-cold RIPA buffer and centrifuged at 12,000 × g (10 min; 4°C) and an aliquot of the supernatant was used for determining protein content. This RIPA-*soluble* fraction was immunodepleted (35) of any FL-APP by immunoprecipitation with the C-terminally-directed antibody and then sequentially immunoprecipitated using the 6E10 antibody (to isolate any Aβ peptide fragments); the resulting immunocomplex was resolved on a discontinuous 8 M urea gel system (36). The 6E10 and 4G8 signals were detected using IRDye^®^ 800CW Goat anti-Mouse IgG (H + L) [LICOR 925-32210] and densitometry was performed using supporting LICOR software.

The pellets leftover after the initial centrifugation in RIPA above were dissolved in 5 M guanidine.HCl (1:20, wt:vol; RT, 2 h). This fraction, e.g., the *insoluble* fraction, was diluted with TBS (1:1, vol:vol) and, as above, Aβ were separated by sequential immunodepletion and immunoprecipitation (36).

### Prefrontal Cortex and Hippocampus Fatty Acid Analysis

Prefrontal cortex and hippocampus tissue from frozen were homogenized in Tris-HCL buffer and transferred into Kimax tubes, where total lipids were then extracted using 2:1 ratio of chloroform/methanol (37). Tissue samples were then spiked with tridecanoic acid (13:0) to act as an internal standard and methylated (38). Fatty acid profile was acquired through gas chromatography as previously described (39). Fatty acid methyl esters were separated on a UFM-RTX WAX analytical column (Thermo Electron Corp., Milan, Italy) using gas chromatography (Trace GC Ultra, Thermo Electron Corp, Milan, Italy), that had been fitted with a fast-flame ionization detector, a split-splitless injector, and Triplus AS autosampler (39, 40). Fatty acids were identified by retention time as compared with known standards (Supelco 37 component FAME mix, Supelco, Bellefonte, PA).

### Statistical Analyses

Cohen's d effect sizes were calculated (41–43) to determine the magnitude and direction of change in protein markers and lipid profiles. Differences between protein markers and lipid profiles were analyzed using a two-way ANOVA (OVX by AB). Significant interactions were examined with Tukey *post-hoc* analysis. Shapiro-Wilk test was used to check normality, and in the case where data were not normally distributed, data was logarithmically transformed. All data are presented as mean ± SEM, with significance reported as *p* ≤ 0.05 and *p*-values approaching significance, i.e., *p* = 0.06–0.10, reported as trends. The Pearson correlation coefficient for protein markers and average memory scores were determined and only significant correlations are reported.

## Results

### Hemodynamic Characteristics and Cognitive Function

A comprehensive cardiac phenotype for the exact same animals used in this study is available in Table 1 and Figure 1 from Olver et al. (11). Briefly, the EF% was normal (i.e., all groups >50%), cardiac output, blood pressure and body mass were similar among groups (*p* ≥ 0.15). Cardiac pressure overload increased heart weight:body mass ratio as well as left ventricle brain-natriuretic peptide mRNA (biomarker for HF) (main effect of AB *p* < 0.05), indicative of HF with compensated resting systolic function. The loss of female sex hormones was coupled with increased total peripheral resistance (main effect of OVX *p* < 0.05) as well as decreased index of cerebral blood flow (main effect of OVX *p* < 0.05), which was most pronounced in the AB-OVX group (*post-hoc, p* < 0.05). Similarly, the loss of female sex hormones coincided with impaired reference (surrogate for long-term memory) and working memory (surrogate for short-term memory) scores (main effect of OVX *p* < 0.05), with impairments being most pronounced in the AB-OVX group (*post-hoc, p* < 0.05). Overall, the data indicate the loss of female sex hormones alone reduces brain blood flow and impairs cognition, and such effects are exacerbated in the setting of cardiac pressure overload.

### Neural Signaling

**Figure 2:**
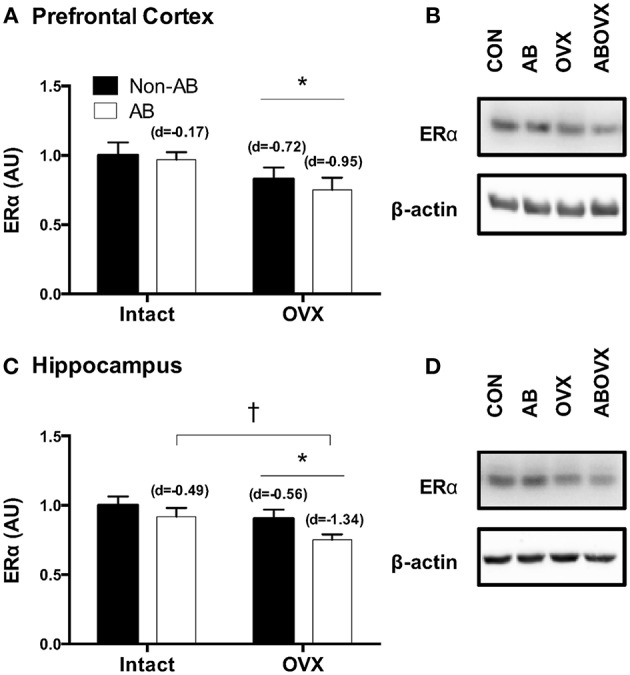
**(A)** ERα prefrontal cortex, **(B)** representative blots, **(C)** ERα hippocampus, and **(D)** representative blots. Data analyzed using a two-way ANOVA. ^*^Main effect of OVX (*p* ≤ 0.03); ^†^trend toward a main effect of AB (*p* = 0.06). d = Cohen's d effect size (small = 0.20, medium = 0.50, large = 0.80); OVX, ovariectomy; AB, aortic banding.

**Figure 3:**
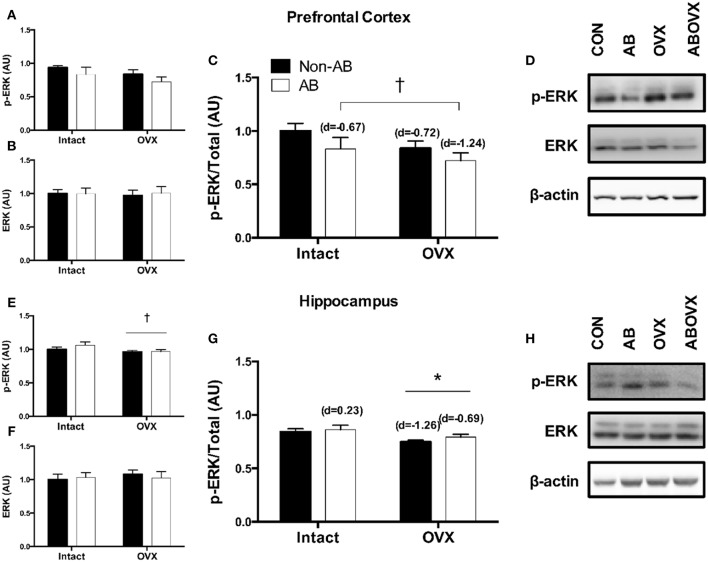
**(A)** p-ERK prefrontal cortex, **(B)** total ERK prefrontal cortex, **(C)** p-ERK:ERK ratio prefrontal cortex, **(D)** representative blots for prefrontal cortex, **(E)** p-ERK hippocampus, **(F)** total ERK hippocampus, **(G)** p-ERK:ERK ratio hippocampus, and **(H)** representative blots for hippocampus. Data analyzed using a two-way ANOVA. ^*^Main effect of OVX (*p* < 0.01); ^†^trend toward a main effect of AB (*p* = 0.08). d = Cohen's d effect size (small = 0.20, medium = 0.50, large = 0.80); OVX, ovariectomy; AB, aortic banding.

The loss of female sex hormones was associated with reduced ERα content in both the prefrontal cortex and the hippocampus (main effect of OVX: *p* ≤ 0.05; Figures 2A–D). Further, cardiac pressure overload was associated with a trend toward reduced ERα content in the hippocampus alone (main effect of AB: *p* = 0.06; Figure 2C). Effect size analysis revealed the loss of female sex hormones in combination with cardiac pressure overload elicited the greatest magnitude of change, indicated by a large, negative effect on ERα content in both brain regions (Figure 2). Levels of total ERK in the prefrontal cortex and hippocampus were similar among groups (*p* ≥ 0.56; Figures 3B,F,D,H). However, cardiac pressure overload and the loss of female sex hormones appeared to affect ERK activation in a region-specific manner (Figures 3A–H). Specifically, cardiac pressure overload was associated with a trend toward a reduced ratio of p-ERK:ERK in the prefrontal cortex (main effect of AB; *p* = 0.08; Figure 3C) and the loss of female sex hormones was associated with reduced ratio of p-ERK:ERK in the hippocampus (main effect of OVX; *p* < 0.01; Figure 3G). Effect size analysis indicated that the loss of female sex hormones combined with cardiac pressure overload resulted in the greatest magnitude of change in the prefrontal cortex, indicated by a large, negative effect on p-ERK:ERK. However, whereas cardiac pressure overload had a medium, positive effect on p-ERK:ERK in the hippocampus, the loss of female sex hormones alone elicited a greater and opposite change, indicated by a large, negative effect on p-ERK:ERK (Figure 3).

**Figure 4:**
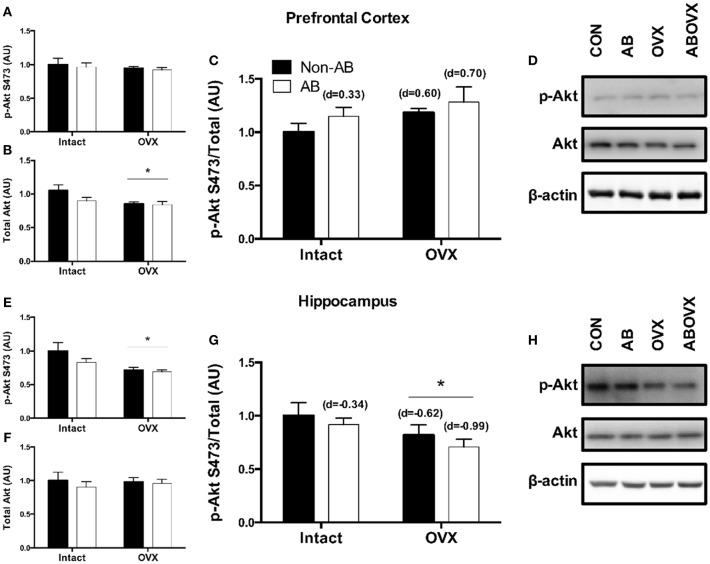
**(A)** p-Akt Ser473 prefrontal cortex, **(B)** total Akt prefrontal cortex, **(C)** p-Akt:Akt ratio prefrontal cortex, **(D)** representative blots for prefrontal cortex, **(E)** p-Akt Ser473 hippocampus, **(F)** total Akt hippocampus, **(G)** p-Akt:Akt ratio hippocampus, and **(H)** representative blots for hippocampus. Data analyzed using a two-way ANOVA. ^*^Main effect of OVX (*p* = 0.03). d = Cohen's d effect size (small = 0.20, medium = 0.50, large = 0.80); OVX, ovariectomy; AB, aortic banding.

The loss of female sex hormones was associated with reduced total Akt in the prefrontal cortex (main effect of OVX: *p* = 0.03; Figures 4A,B,D). However, the ratio of p-Akt Ser473:Akt in the prefrontal cortex was similar among groups (*p* ≥ 0.11; Figures 4C,D). In contrast, total Akt in the hippocampus was similar among groups (*p* ≥ 0.35; Figures 4F,H), but the loss of female sex hormones was associated with reduced ratio of p-Akt:Akt (main effect of OVX: *p* = 0.04; Figures 4E,G,H). Effect size analysis revealed the loss of female sex hormones in combination with cardiac pressure overload elicited the greatest magnitude of change, evidenced by a medium, positive effect on p-Akt:Akt in the prefrontal cortex and a large, negative effect on p-Akt:Akt in the hippocampus (Figures 4C,G).

**Figure 5:**
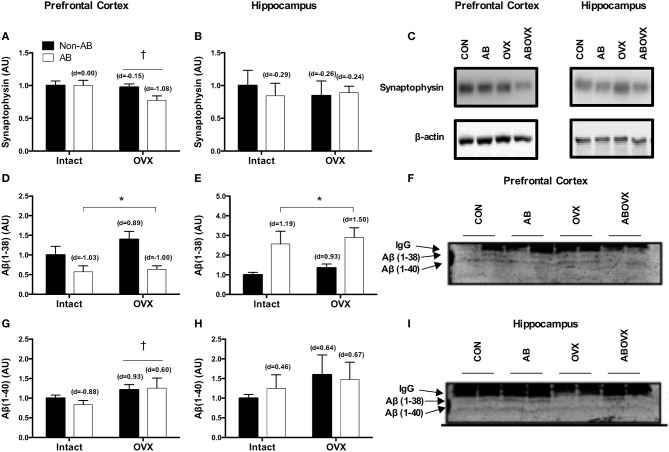
Synaptophysin content in the **(A)** prefrontal cortex, **(B)** hippocampus and a **(C)** representative blot; Aβ(1–38) in the **(D)** prefrontal cortex **(E)** hippocampus and a **(F,I)** representative blot; Aβ (1–40) in the **(G)** prefrontal cortex, **(H)** hippocampus and a **(F,I)** representative blot. Data analyzed using a two-way ANOVA. ^†^Trend toward a main effect of OVX (*p* = 0.07–0.09). ^*^Main effect of AB (*P* < 0.05). d = Cohen's d effect size (small = 0.20, medium = 0.50, large = 0.80); OVX, ovariectomy; AB, aortic banding; IgG, light chain of the antibody used for immunoprecipitation.

The loss of female sex hormones was associated with a trend toward a reduction in levels of synaptophysin, a synaptic vesicle membrane protein and marker of synaptic plasticity, in the prefrontal cortex (main effect of OVX: *p* = 0.07; Figures 5A,C). However, no changes were observed in the hippocampus for synaptophysin (*p* ≥ 0.61; Figures 5B,C). Effect size analysis indicated the loss of female sex hormones in combination with cardiac pressure overload elicited a large, negative effect on synaptophysin levels in the prefrontal cortex (Figure 5A). Cardiac pressure overload was associated with decreased Aβ(1–38) content in the prefrontal cortex and increased Aβ(1–38) content in the hippocampus (main effect of AB; *p* < 0.01; Figures 5D–F,I). The loss of female sex hormones alone resulted in large, positive effect on Aβ(1–38) content in both regions. However, cardiac pressure overload alone and in conjunction with the loss of female sex hormones elicited regional-specific changes, indicated by a large, negative effect on Aβ(1–38) content in the prefrontal cortex and a large, positive effect on Aβ(1–38) content in the hippocampus. The loss of female sex hormones was associated with a trend toward increased Aβ(1–40) content in the prefrontal cortex (main effect of OVX; *p* = 0.09; Figures 5F,G), but neither intervention had a significant effect on Aβ(1–40) in the hippocampus (*p* ≥ 0.31; Figures 5H,I). Effect size analysis revealed the loss of female sex hormones alone and in conjunction with cardiac pressure overload resulted in a large and medium, positive effect, respectively, on Aβ(1–40) content in the prefrontal cortex. There were no differences in p-GSK-β, pSer396-Tau, or IDE in either the prefrontal cortex or hippocampus among groups (*p* ≥ 0.12) and Aβ(1–42) protein was only detected in a sub-set of samples and not consistently in any treatment group (*data not shown*). Furthermore, we were unable to detect any Aβ peptide in any of the insoluble/plaque associated fractions tested (*data not shown*).

### Brain Lipid Content

**Figure 6:**
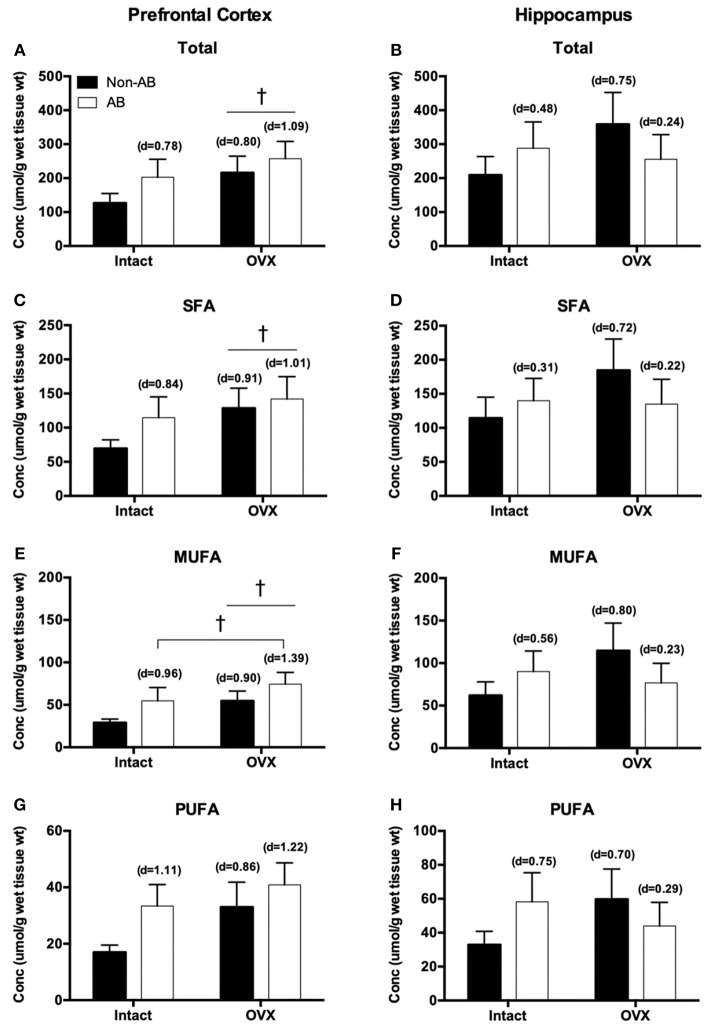
Total fatty acids in the **(A)** prefrontal cortex and **(B)** hippocampus; saturated fatty acids (SFA) in the **(C)** prefrontal cortex and **(D)** hippocampus; monounsaturated fatty acids (MUFA) in the **(E)** prefrontal cortex and **(F)** hippocampus; polyunsaturated fatty acids (PUFA) in the **(G)** prefrontal cortex and **(H)** hippocampus. Data analyzed using a two-way ANOVA. ^†^Trend toward a main effect of OVX (*p* = 0.06–0.10). d = Cohen's d effect size (small = 0.20, medium = 0.50, large = 0.80); OVX, ovariectomy; AB, aortic banding.

The fatty acid (FA) profile for both the prefrontal cortex and hippocampus are summarized in Table 1. In the prefrontal cortex, there were no statistically significant effects of either intervention on lipid content. However, the loss of female sex hormones was associated with a trend toward increased total, saturated and monounsaturated fatty acid content (main effect OVX; *p* = 0.07–0.10; Figures 6A,C,E), with no differences in polyunsaturated fatty acid content (*p* = 0.15; Figure 6G). Despite there being no changes in polyunsaturated fatty acid content, the loss of female sex hormones was associated with an increased ratio of n3/n6 fatty acids (main effect of OVX; *p* = 0.01; control = 1.8 ± 0.41, AB = 2.8 ± 0.6, OVX = 4.2 ± 1.1, AB + OVX = 3.5 ± 0.5). Cardiac pressure overload was associated with a trend toward increased monounsaturated fatty acid content (main effect of AB; *p* = 0.07) with no differences in total, saturated and polyunsaturated fatty acid content (*p* ≥ 0.13). Effect size analysis revealed the loss of female sex hormones, in combination with cardiac pressure overload, resulted in the greatest magnitude of increase relative to the control group, evidenced by a large, positive effect on total, saturated, monounsaturated and polyunsaturated lipid content in the prefrontal cortex. In the hippocampus, there were no significant effects of either intervention on the lipid profile, including the n3/n6 ratio (*p* ≥ 0.17; Figures 6B,D,F,H). Effect size analysis indicated the loss of female sex hormones had a medium, positive effect on total, saturated, monounsaturated and polyunsaturated fatty acid content and cardiac pressure overload alone had a medium, positive effect on mono- and polyunsaturated fatty acid content in the hippocampus.

**Table 1:** Fatty acid species concentration.

**Fatty acids**	**Region**	**CON**	**AB**	**OVX**	**AB-OVX**
12:0	PFC	0.07 ± 0.01	0.08 ± 0.01	0.16 ± 0.05	0.12 ± 0.03
	HC	0.09 ± 0.02	0.15 ± 0.05	0.17 ± 0.04	0.12 ± 0.03
14:1	PFC	0.16 ± 0.02	0.21 ± 0.03	0.23 ± 0.04	0.20 ± 0.05
	HC	0.16 ± 0.03	0.18 ± 0.04	0.27 ± 0.06	0.23 ± 0.02
15:0	PFC	0.18 ± 0.04	0.19 ± 0.05	0.22 ± 0.04	0.27 ± 0.06
	HC	0.24 ± 0.07	0.30 ± 0.08	0.44 ± 0.10	0.27 ± 0.09
15:1	PFC	0.29 ± 0.09	1.06 ± 0.30	0.86 ± 0.15	1.12 ± 0.34
	HC	0.75 ± 0.26	1.12 ± 0.35	2.64 ± 0.95	0.81 ± 0.27
16:1	PFC	2.11 ± 0.29	4.02 ± 1.21	4.10 ± 0.88	5.54 ± 1.06
	HC	4.37 ± 1.19	5.16 ± 1.36	7.75 ± 2.21	4.98 ± 1.53
17:0	PFC	0.47 ± 0.10	0.61 ± 0.14	0.70 ± 0.18	1.01 ± 0.19
	HC	0.72 ± 0.17	0.86 ± 0.26	1.07 ± 0.32	1.00 ± 0.26
17:1	PFC	1.79 ± 0.05	2.21 ± 0.59	2.01 ± 0.35	3.52 ± 0.81
	HC	2.13 ± 0.61	4.72 ± 2.07	3.77 ± 1.01	3.29 ± 0.96
18:0	PFC	28.54 ± 5.81	46.24 ± 12.28	51.05 ± 12.14	58.98 ± 12.62
	HC	47.57 ± 12.28	61.47 ± 16.51	84.31 ± 20.91	59.49 ± 16.73
18:1	PFC	22.87 ± 3.26	45.58 ± 13.03	45.52 ± 9.97	59.89 ± 11.49
	HC	51.07 ± 13.21	74.49 ± 20.06	95.68 ± 26.94	63.51 ± 19.34
18:2n6	PFC	1.25 ± 0.22	2.17 ± 0.71	2.18 ± 0.49	2.90 ± 0.73
	HC	1.69 ± 0.45	2.43 ± 0.84	2.53 ± 0.73	2.17 ± 0.71
18:3n3	PFC	027 ± 0.15	0.23 ± 0.07	0.33 ± 0.10	1.51 ± 0.78
	HC	0.19 ± 0.06	0.39 ± 0.16	0.52 ± 0.17	0.41 ± 0.17
20:0	PFC	0.50 ± 0.32	0.39 ± 0.14	0.44 ± 0.12	0.61 ± 0.24
	HC	0.51 ± 0.15	0.61 ± 0.23	0.96 ± 0.35	0.81 ± 0.21
20:1	PFC	1.18 ± 0.46	1.39 ± 0.47	1.39 ± 0.47	3.78 ± 1.67
	HC	2.33 ± 0.73	3.83 ± 1.02	3.76 ± 1.15	3.15 ± 1.53
20:2n6	PFC	0.29 ± 0.06	0.99 ± 0.33	0.81 ± 0.27	0.86 ± 0.15
	HC	0.73 ± 0.12	1.98 ± 0.76	1.71 ± 0.51	1.27 ± 0.61
20:3n6	PFC	1.73 ± 0.62	3.01 ± 0.74	2.11 ± 0.84	4.11 ± 1.93
	HC	1.64 ± 0.45	7.49 ± 4.64	3.46 ± 1.15	4.31 ± 2.04
22:2n6	PFC	0.38 ± 0.20	0.65 ± 0.27	0.33 ± 0.17	0.67 ± 0.25
	HC	0.69 ± 0.17	1.02 ± 0.43	1.67 ± 0.64	0.93 ± 0.42
23:0	PFC	2.98 ± 0.23	6.57 ± 2.17	7.18 ± 2.07	7.38 ± 1.92
	HC	7.31 ± 2.18	9.16 ± 2.81	11.21 ± 2.85	7.73 ± 2.11
n3	PFC	12.16 ± 1.45	24.94 ± 6.78	26.00 ± 7.41	31.08 ± 5.80
	HC	27.72 ± 7.12	43.28 ± 12.62	48.28 ± 14.75	34.32 ± 10.46
n6	PFC	6.04 ± 1.22	8.45 ± 1.11	6.95 ± 1.80	9.82 ± 2.37
	HC	5.16 ± 1.04	14.87 ± 5.72	11.40 ± 3.22	9.52 ± 3.66

*Values are represented as means ± SEM; Units, μmol/g wet wt.; PFC, prefrontal cortex; HC, hippocampus; CON, control or intact; AB, aortic banding; OVX, Ovariectiomy*.

### Correlations With Cognitive Function

**Figure 7:**
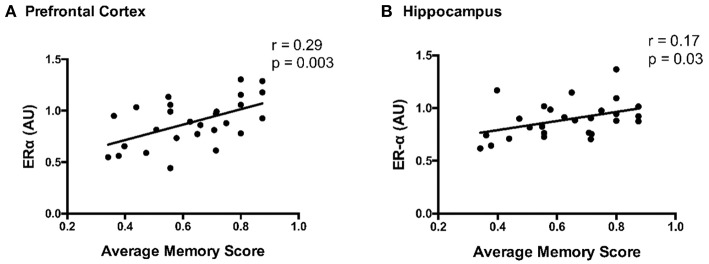
Correlation between ERα content and average memory scores in the **(A)** prefrontal cortex and **(B)** hippocampus; Pearson correlation coefficient = *r*.

ERα content and average memory scores were positively correlated in both the prefrontal cortex and hippocampus (*p* ≤ 0.03; Figure 7). Correlations between other protein markers and average memory were not significant (*p* ≥ 0.10).

## Discussion

In this study, we provide novel insight into how the loss of female sex hormones (OVX) and HF (AB) independently contribute to altered cell signaling in the prefrontal cortex and the hippocampus in a pre-clinical swine model of cardiogenic dementia. In the context of post-menopausal HF (i.e., AB-OVX group) related dementia, our findings indicate region-dependent profiles. For example, there is a potential role for reduced ERα content, decreased ERK signaling, and decreased Aβ(1–38) content in the prefrontal cortex that corresponded with a loss of synaptophysin content and increased lipid content. In contrast, hippocampal extracts revealed a similar loss of ERα content, with a reduction in ERK signaling and the pro-survival kinase Akt, with no obvious effect on synaptophysin content or lipid content. Earlier work from these same animals suggests cerebrovascular dysfunction is a key characteristic of the cardiogenic dementia profile (11). Thus, in aggregate, the data indicate cardiogenic dementia is associated with both cerebrovascular and neuronal changes that promote impairments in brain blood flow regulation and alterations in proteins and key kinases involved in the encoding and retrieval of memories, synaptic plasticity, and neuronal survival in the prefrontal cortex and hippocampus (23, 25–28).

The current data suggest the loss of female sex hormones and HF independently contribute to the cardiogenic dementia profile in this swine model of HF. Our previous work in swine (11) and other's work in rodents (44–46) demonstrate the loss of female sex hormones results in impairments in spatial navigation and spatial memory. The current data extend on earlier findings and suggest impairments in spatial memory are associated with reduced ERα content in the prefrontal cortex and hippocampus. Further, mirroring the earlier observation that memory deficits were most pronounced in AB-OVX swine (11), the current data show reductions in ERα content of the hippocampus, a brain region involved in encoding and retrieval of memories (13), tended to be greatest in AB-OVX swine. The ERα is a key regulator of kinase activity and serves a complex role in memory (47, 48). Given the current data highlight region-specific effects of the loss of female sex hormones and cardiac pressure overload on ERα content in the brain, it is possible hormonal signaling plays a pivotal role in the cardiogenic dementia profile of post-menopausal HF patients.

Two key signaling cascades implicated in the recovery and programming of memories that are also influenced by ERα-activation of tyrosine kinase receptors are Akt and ERK (23, 25–28, 47). In the current study, cardiac pressure overload had minimal effect on Akt in either brain region, but tended to reduce ERK signaling in the prefrontal cortex. In contrast, the loss of female sex hormones was associated with reduced total Akt in the prefrontal cortex and reduced Akt signaling in the hippocampus. Furthermore, the loss of female sex hormones decreased ERK signaling in the hippocampus. The aforementioned data indicate reductions in Akt and ERK signaling following the loss of female sex hormones occur preferentially in the hippocampus compared with the prefrontal cortex in this model. Targeted changes in the hippocampus support earlier observations in OVX swine that reference memory and spatial navigation (11), both hippocampal dominant processes (13), were impaired following the loss of female sex hormones. From a clinical perspective, the current data suggest the loss of female sex hormones and cardiac pressure overload contribute directly to the cardiogenic dementia profile in a brain region-specific manner, independent of resting cardiac systolic impairment.

Despite changes in ERα content, ERK, and Akt signaling affecting the AB-OVX group, differences in downstream targets p-GSK-3β, pSer396-Tau, or IDE were not significant nor was the longer, more hydrophobic and AD-related Aβ(1–42) peptide detected consistently. In keeping with the observation that immunoprecipitation strategies did not reveal any Aβ peptide in the insoluble (aggregation/plaque-associated) fraction of these cortical and hippocampal samples, these findings might reflect the fact that the animals have maintained normal transport mechanisms for removal of the Aβ(1–42) peptides into the circulation. In addition, these findings may also reflect that animals were examined during a developmental stage of cardiogenic dementia, prior to overt changes in GSK and the subsequent development of tau pathology (49, 50). Aging is a primary cause of dementia (51) and it is impractical to age pigs to true senescence for pre-clinical research given their relatively long life span (~15 years) (52). Of note, tau pathology may develop after Aβ aggregation and deposition, which can occur 10–20 years before the onset of clinical symptoms (16, 50, 53, 54). In this regard, the changes observed in the current study may reflect early pathological adaptations in disease progression.

Consistent with the latter interpretation, synaptophysin content, a marker for synaptic plasticity/density, tended to be reduced in the prefrontal cortex following the loss of female sex hormones with the greatest reduction in the AB-OVX group. The parallel loss of frontocortical ERα expression, ERK signaling, and Aβ(1–38) content and a tendency for increase in Aβ(1–40) content in the AB-OVX group may also be quite relevant. Indeed, ERK is known to influence memory (55) as well as shift away from the non-amyloidogenic (56) generation of N-terminally truncated p3 fragment (an effect we observed preliminarily using the 4G8 epitope, *data not shown*), in favor of longer, more hydrophobic Aβ variants such as Aβ(1–40), and all in an ER-dependent manner. All of these molecular phenotypes would suggest a generalized, dysfunctional phenotype in this brain region. The latter finding is consistent with earlier work that shows OVX increases Aβ(1–40) in a transgenic mouse model of AD (57), highlighting a possible protective effect of female sex hormones or ER-α signaling against Aβ(1–40) accumulation. There is emerging interest in the interactive role and clinical utility of Aβ variants such as the 38-/40-mers and their processing in the pathogenesis of multiple dementia phenotypes (58–61). Available evidence indicates that amyloid plaques containing the 38/40-mers are prominent in several forms of dementia and the presence or ratio of multiple Aβ variants may alter the behavior and toxicity of the entire peptide pool (58–60, 62). Thus, the current data highlight that both heart failure and the loss of female sex hormones may influence the cardiogenic dementia profile by altering the Aβ pool in a region-dependent manner without necessarily producing an overt AD-like phenotype [i.e., increased Aβ(1–42)].

In the current study, neither intervention significantly altered the brain lipid profile. However, similar to reductions in ERα content and ERK signaling, the accumulation of total, saturated and monounsaturated fatty acids in the prefrontal cortex tended to be more apparent following the loss of female sex hormones. Further, the magnitude of change was greatest in the combined AB-OVX group. Regarding this finding, ERα may play a direct role in lipid status in the brain by directly modulating lipid transport as well as enzymes involved in fatty acid synthesis and oxidation (21–23). Why such trends were only observed in the prefrontal cortex and changes following the loss of female sex hormones appeared to be potentiated by cardiac pressure overload remains unknown. Other mechanisms, such as enhanced blood brain barrier permeability may also be involved (21, 63, 64). Recently, it was reported that OVX increased blood brain barrier permeability in rats (63), possibly the result of elevated barrier inflammation (64). Cerebrovascular inflammation was not examined in the current study, but earlier work highlights that the loss of female sex hormones resulted in cerebrovascular dysfunction with the most pronounced impairments occurring in the combination group (11), suggesting cerebrovascular contributors may be implicated. Lipid metabolism is highly regulated and involved in the maintenance of neuronal structure and function within the brain (21, 65, 66). Although it remains unclear how alterations in the brain lipid profile affect these actions specifically, the present analyses provide an important first step toward characterizing regional changes that may precipitate the development of cardiogenic dementia.

This study provides evidence that the loss of female sex hormones and cardiac pressure overload independently affect ERα content, Akt, and ERK signaling as well as Aβ peptide content and lipid content in a region-specific manner in the prefrontal cortex and hippocampus in swine. Such changes manifested without advanced aging or resting cardiac systolic impairment, highlighting direct roles for menopause and HF (with normal EF%) in the cardiogenic dementia profile. Combined with previous work from these animals (11) and in line with human data (9, 14), it appears cardiogenic dementia involves both cerebrovascular and neural maladaptations. Determining whether these maladaptations precipitate and contribute to the presentation of cognitive dysfunction is critical to better understanding and developing effective therapies for cardiogenic dementia.

## Data Availability

All datasets generated for this study are included in the manuscript/supplementary files.

## Ethics Statement

This was a retrospective analysis on tissues harvested from a previous study where all experimental and animal protocols were approved by the University of Missouri (Columbia, MO) Animal Care and Use Committee.

## Author Contributions

GH and TO composed the manuscript. All authors contributed to the data collection, analyses, and edited the manuscript.

### Conflict of Interest Statement

The authors declare that the research was conducted in the absence of any commercial or financial relationships that could be construed as a potential conflict of interest.
